# Measurement of islet cell antibodies in the Type 1 Diabetes Genetics Consortium: efforts to harmonize procedures among the laboratories

**DOI:** 10.1177/1740774510373496

**Published:** 2010-08

**Authors:** Polly J Bingley, Alistair JK Williams, Peter G Colman, Shane A Gellert, George Eisenbarth, Liping Yu, Letitia H Perdue, June J Pierce, Joan E Hilner, Concepcion Nierras, Beena Akolkar, Michael W Steffes, the T1DGC

**Affiliations:** ^a^Department of Clinical Science at North Bristol, University of Bristol, Bristol, UK, ^b^Departments of Diabetes and Endocrinology and Pathology, Royal Melbourne Hospital, Melbourne, Victoria, Australia, ^c^Barbara Davis Center for Childhood Diabetes, University of Colorado Health Sciences, Denver, CO, USA, ^d^Division of Public Health Sciences, Wake Forest University Health Sciences, Winston-Salem, NC, USA, ^e^Department of Biostatistics, School of Public Health, University of Alabama at Birmingham, Birmingham, AL, USA, ^f^Juvenile Diabetes Research Foundation International, New York, NY, USA, ^g^Division of Diabetes, Endocrinology and Metabolic Diseases, National Institute of Diabetes and Digestive and Kidney Diseases, National Institutes of Health, Bethesda, MD, USA, ^h^Department of Laboratory Medicine and Pathology, University of Minnesota Medical School, Minneapolis, MN, USA

## Abstract

***Background and Purpose*** Three network laboratories measured antibodies to islet autoantigens. Antibodies to glutamic acid decarboxylase (GAD65 [GADA]) and the intracellular portion of protein tyrosine phosphatase (IA-2_ic_ [IA-2A]) were measured by similar, but not identical, methods in samples from participants in the Type 1 Diabetes Genetics Consortium (T1DGC).

***Methods*** All laboratories used radiobinding assays to detect antibodies to *in vitro* transcribed and translated antigen, but with different local standards, calibrated against the World Health Organization (WHO) reference reagent. Using a common method to calculate WHO units/mL, we compared results reported on samples included in the Diabetes Autoantibody Standardization Program (DASP), and developed standard methods for reporting in WHO units/mL. We evaluated intra-assay and inter-assay coefficient of variation (CV) in blind duplicate samples and assay comparability in four DASP workshops.

***Results*** Values were linearly related in the three laboratories for both GADA and IA-2A, and intra-assay technical errors for values within the standard curve were below 13% for GADA and below 8.5% for IA-2A. Correlations in samples tested 1–2 years apart were >97%. Over the course of the study, internal CVs were 10–20% with one exception, and the laboratories concordantly called samples GADA or IA-2A positive or negative in 96.7% and 99.6% of duplicates within the standard curve. Despite acceptable CVs and general concordance in ranking samples, the laboratories differed markedly in absolute values for GADA and IA-2A reported in WHO units/mL in DASP over a large range of values.

***Limitations*** With three laboratories using different assay methods (including calibrators), consistent values among them could not be attained.

***Conclusions*** Modifications in the assays are needed to improve comparability of results expressed as WHO units/mL across laboratories. It will be essential to retain high intra- and inter-assay precision, sensitivity and specificity and to confirm the accuracy of harmonized methods.

## Introduction

The Type 1 Diabetes Genetics Consortium (T1DGC) comprises groups of investigators from many countries throughout the world, with a common goal of identifying genes predisposing to type 1 diabetes mellitus. Three T1DGC network laboratories (in Asia-Pacific, Europe, and North America) were selected to measure antibodies to the islet autoantigens: glutamic acid decarboxylase (GAD65 [GADA]) and the intracellular portion of protein tyrosine phosphatase (IA-2_ic_ [IA-2A]) as part of the determination of phenotypes for the project [1–5]. Autoantibodies were measured in samples from all T1DGC participants with type 1 diabetes. Although the measurement was not used as an entry criterion for participation in the study, the research value of quantifying results in standardized World Health Organization (WHO) units/mL to allow more detailed phenotyping became apparent during the early stages of planning; *i.e.*, that continuous values would permit additional analysis in relating genotypes to phenotypes.

This article describes the methods used in these laboratories, and the quality control procedures to maintain and monitor the performance of each laboratory. A masked split duplicate program allowed assessment of intra- and inter-assay reproducibility over time for each of the assays, including assessment of different methods of computing results reported in WHO units/mL for sera yielding signals above the highest WHO standard. The results of the Diabetes Autoantibody Standardization Program (DASP) for the three laboratories are also presented. The DASP workshops aim to improve and standardize measurement of autoantibodies associated with type 1 diabetes among the laboratories, and performance in DASP was used as a criterion for selecting the laboratories and for monitoring their performance [6,7]. Finally, we summarize the decisions taken regarding the assay procedures and reporting of results to bring the laboratories into closer alignment.

## Methods

Given the international nature of the T1DGC and the extended distances that it covered, there was a clear need to establish regional laboratories, and three laboratories were selected on the basis of performance in DASP, a program organized by the Immunology of Diabetes Society and the Centers for Disease Control and Prevention. These laboratories have interacted for years (through DASP and other programs), using radiobinding assays with a generally similar format [8–10], but some differences as shown in Table 1. The following sections summarize the main similarities and differences.

**Table 1:** Comparison of characteristics of the assays in the T1DGC laboratories

	Asia-Pacific	European	North American
Assay format	Radiobinding assay in 96-well filtration plate	Radiobinding assay in 96 deep-well plate	Radiobinding assay in 96-well filtration plate
Buffer	5 mmol/L Tris, 150 mmol/L NaCl, 1 mmol/L L-methionine, 0.1% (w/v) BSA, 1% (v/v) Tween 20, pH 7.4	50 mmol/L Tris, 150 mmol/L NaCl, 1% (v/v) Tween 20, pH 7.4	20 mmol/L Tris, 150 mmol/L NaCl, 0.1% (w/v) BSA, 0.1% sodium azide, 0.15% (v/v) Tween 20, pH 7.4
GADA plasmid	Full length	Full length	Full length (PEX9)
	E. Bonifacio	E. Bonifacio	A. Lernmark
IA-2A plasmid	604–979	606–979	604–979
	E. Bonifacio	M. Christie	E. Bonifacio
Radiolabel	^35^S-methionine (GADA and IA-2A)	^35^S-methionine (GADA and IA-2A)	^3^H-leucine (GADA), 35-S methionine (IA-2A)
	30,000 cpm/well in 50 µL	15,000 cpm/well in 25 µL	20,000 cpm/well in 50 µL
Buffer	5 mmol/L Tris, 150 mmol/L NaCl, 1 mmol/L L-methionine, 0.1% (w/v) BSA, 1% (v/v) Tween 20, pH 7.4	50 mmol/L Tris, 150 mmol/L NaCl, 1% (v/v) Tween 20, pH 7.4	20 mmol/L Tris, 150 mmol/L NaCl, 0.1% (w/v) BSA, 0.1% sodium azide, 0.15% (v/v) Tween 20, pH 7.4
Primary incubation	5 µL serum in duplicate, 16 h at 4°C	2 µL serum in duplicate, 20 h at 4°C	2 µL serum in duplicate, 20 h at 4°C
Separation and washing	5 µL/well PAS in 50 µL incubated for 1 h, washed by vacuum filtration	5 µL/well PAS in 50 µL incubated 1.5 h, washed by centrifugation/aspiration	12.5 µL/well PAS in 25 µL incubated 0.75 h, washed by vacuum filtration

### Standards

Each laboratory had prepared local standards calibrated to the WHO international reference reagent for GADA and IA-2A antibodies [11] used over the course of the DASP workshops [6]. The Asia-Pacific laboratory collected a serum sample from a patient with Stiff Person Syndrome (who was highly positive for both GADA and IA-2A); the European laboratory used sera from islet cell antibody-positive relatives of patients with type 1 diabetes; and the North American laboratory pooled sera from type 1 diabetes patients and GADA/IA-2A positive relatives.

### Labeled clones

All laboratories used similar clones to prepare target antigens for both antibodies. For the GADA antibody assay, both the Asia-Pacific and European laboratories used a clone from the same source (Ezio Bonifacio, Milan, Italy), while the North American laboratory used a different clone (Åke Lernmark, Seattle, WA, USA). For the IA-2A antibody assay, the Asia-Pacific and North American labs used a clone from the same source (Ezio Bonifacio, Milan, Italy), while the European laboratory used a different clone (Michael Christie, London, UK). All laboratories used similar transcription/translation kits (Promega, Madison, WI, USA) to produce labeled GADA and IA-2A, followed by removal of unincorporated label using gel exclusion chromatography. The Asia-Pacific and European laboratories labeled both the GADA and IA-2A proteins with ^35^S-methionine. The North American laboratory labeled the IA-2A protein with ^35^S-methionine and the GADA protein with ^3^H-leucine to allow both antibodies to be measured in a single, combined assay.

The similarity of labeling methods used would be expected to result in the production of qualitatively similar labeled proteins for each assay among the laboratories. There may, however, be considerable differences in amounts of proteins actually present in the labeled material, both within a single laboratory over several different labeling processes and between laboratories, as a result of differences in the specific activities of the labeled proteins produced and the duration of storage. Indeed, the amounts of sera and label used varied among the laboratories (Table 1).

### Separation

The laboratories adopted similar, but not identical, procedures to remove the unbound labeled protein using protein-A sepharose to bind to the antibody–antigen complex. The Asia-Pacific and North American laboratories formed immunocomplexes in wells in filter plates; to remove any unbound labeled protein, washes were added to the plate and removed by vacuum filtration. The North American laboratory performed two sets of four washes, with a 5 min shake between each set, while the Asia-Pacific laboratory performed 10 washes. Both laboratories counted the filter plates. The European laboratory formed immunocomplexes in wells in deep-well plates and removed the unbound labeled protein by five cycles of wash, centrifugation, and aspiration, and transferred the pellets to another plate for counting in a TopCount β-counter (Perkin Elmer Life and Analytical Sciences Inc, Waltham, MA, USA).

### Standard curves and interpolation of values

From the start of the project, the laboratories agreed to calculate results in WHO units/mL derived from a 7-point standard curve, used each time an assay was performed. Values above the highest standard were calculated in two ways:

- as an index related to the highest standard: WHO Units/mL = (value of highest standard) × [(cpm(unknown) – cpm(negative diluent serum))/(cpm(WHO standard) – cpm(negative diluent serum))]
- as WHO units/mL derived by extrapolation of the standard curve.

The European and North American laboratories used a logarithmic curve fit for calculating values (Excel, Microsoft), while the Asia-Pacific laboratory used a spline curve fitting program (Multicalc, Packard). These programs caused some differences between laboratories, particularly for extrapolated values above the range of the standard curve (data not shown).

### Thresholds

Each laboratory defined its own threshold for calling samples positive or negative for the purposes of the study. The threshold for the European laboratory was set as the 97.5th percentile of 2860 schoolchildren expressed in WHO units/mL [9]. The North American laboratory cut-points were set at indices of 0.032 for GADA autoantibodies and 0.049 for IA-2A autoantibodies, the 99th percentiles of 198 normal controls including children and adults who did not have a first degree relative with diabetes. In Asia-Pacific, the GADA threshold was determined using a receiver operating characteristic (ROC) plot of 246 controls and 137 newly diagnosed patients, with results expressed in local units. The IA-2A threshold was determined using a ROC plot of 145 controls and 49 newly diagnosed patients, in local units. These local laboratory units were used to determine if a sample was positive or negative, but the results were reported in WHO units.

### Quality control procedures

To assess the quality of the measures from the autoantibody laboratories, a two-pronged system was implemented. First, univariate analyses were conducted on the monthly data results uploaded to the Coordinating Center. Within each laboratory, results over time were recorded. Based on these analyses, summary statistics (*e.g.*, means, variances) and out-of-range values were obtained and, if necessary, investigated further. Second, duplicate autoantibody measures were performed on a random sample of approximately 5% of participants with type 1 diabetes. Duplicate sera were collected and labeled with a separate, unique identifier by the clinic staff and were sent to the laboratories in the normal sample shipments. The laboratories were masked as to which samples were paired. These samples were often measured in the same assay and are therefore primarily representative of intra-assay variation. Inter-assay variation was evaluated by a second split duplicate protocol in which previously measured duplicate pairs were resubmitted to each laboratory. The time interval between the initial and second measurements was 1–2 years.

In addition to graphical inspection of the data, reliability was assessed using intraclass correlations and the technical error measurement for autoantibody measures. The technical error is the square root of the pooled between measures variance as a percentage of the sample mean: ((Sqrt(Σ *d*^2^/2*n*))/sample mean) × 100). The technical error was compared to the laboratory’s internal coefficient of variation (CV). If there was evidence of high technical error, the laboratory was contacted and asked for an explanation.

The results of the split duplicates were expressed as antibody positive or negative (as defined within each laboratory) and as WHO units/mL determined both from the standard curves over all values and, when the values were above the highest standard, as an index of the highest value.

## Results

### Intra-assay reproducibility

#### GADA

The technical errors for the Asia-Pacific, European, and North American laboratories were 11.2%, 8.8%, and 12.6%, respectively. The internal CV at low GADA levels were 44.0%, 16.0%, and 18.1%, respectively, and at high levels were 33.0%, 10.0%, and 10.8%. By July 4, 2009, a total of 571 intra-assay split pairs had been tested for GADA: 490 with values in the range of the standard curve and 81 with values above the highest standard. Within the standard curve, the mean difference between the pairs was −0.6 WHO units/mL (standard deviation [SD] 15.1), with 96.7% concordance in positive/negative calls within the pairs. For samples with antibody levels above the highest standard, the mean difference was −31.4 WHO units/mL (SD 241.4), with 100% concordance in positive/negative calls within the pairs.

#### IA-2A

The technical errors for the Asia-Pacific, European, and North American laboratories were 8.5%, 3.4%, and 6.0%, respectively. The internal CVs at low IA-2A levels were 30.0%, 19.0%, and 17.0%, respectively, and at high levels were 30.0%, 20.0%, and 6.7%. By July 4, 2009, a total of 572 intra-assay split pairs had been tested for IA-2A: 479 with values within the range of the standard curve and 93 with values above the highest standard. Within the standard curve, the mean difference between the pairs was −0.04 WHO units/mL (SD 7.9), with 99.6% concordance in positive/negative calls within the pairs. For samples with antibody levels above the top standard, the mean difference was −17.6 WHO units/mL (SD 89.7), with 100% concordance in positive/negative calls within the pairs.

### Inter-assay reproducibility

**Figure 1:**
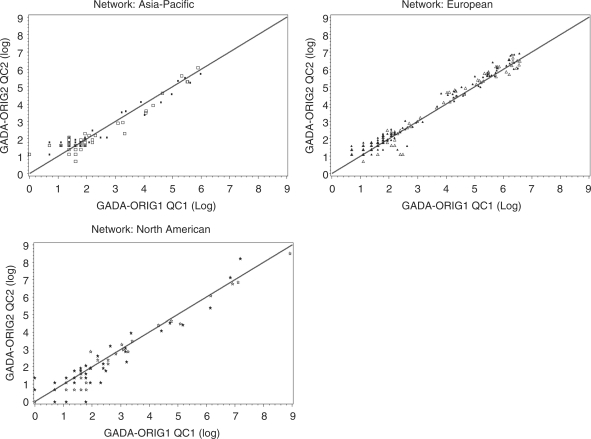
GADA inter-assay comparisons of blind duplicates among the T1DGC autoantibody laboratories. Mean values of the original and repeat assays are plotted. Results above the highest standard have been extrapolated from the standard curve.

**Figure 2:**
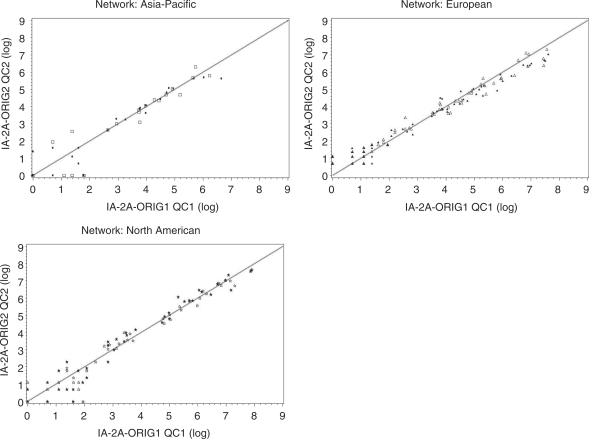
IA-2A inter-assay comparisons of blind duplicates among the T1DGC autoantibody laboratories. Mean values of the original and repeat assays are plotted. Results above the highest standard have been extrapolated from the standard curve.

Many samples (*n* = 384) previously assayed as part of the intra-assay protocol were reassayed 1–2 years later to evaluate inter-assay reproducibility (Figures 1 and 2). Within each laboratory, the assays demonstrated excellent reproducibility, even over this time interval.

### DASP proficiency evaluations

**Figure 3:**
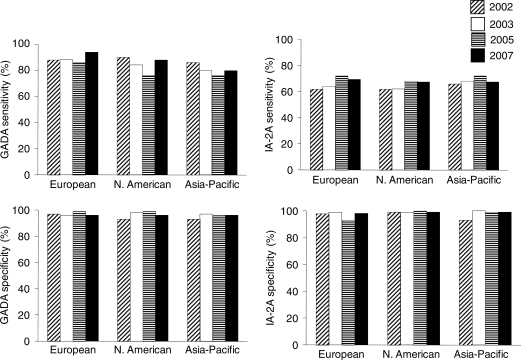
Sensitivity and specificity for GADA (left) and IA-2A (right) in four DASP proficiency evaluations among the three T1DGC autoantibody laboratories. Results are shown for assays using the IA-2_ic_ clone with the exception of the North American DASP 2002 results that used the IA-2_bdc_ clone. A common set of 100 control sera were used for DASP 2002-2005, but 50 were substituted for the 2007 workshop.

All three T1DGC laboratories participated in the four DASP proficiency evaluations conducted since 2000, and during this period achieved levels of sensitivity and specificity, among the best of the participating laboratories (Figure 3) as well as good discrimination between health and disease over time as assessed by ROC curve analysis.

The results of GADA and IA-2A determinations in the fourth DASP proficiency workshop [7] with results expressed in WHO units/mL showed that all three laboratories assigned the same GADA positive/negative status in 43 of 50 samples from patients with newly diagnosed diabetes and 97 of 100 samples from blood donor controls. All three laboratories assigned the same IA-2A positive/negative status in 48 of 50 samples from patients and in 93 of 100 samples from controls. The largest IA-2A discrepancy was in control samples reported as positive that had very low levels of antibody.

**Figure 4:**
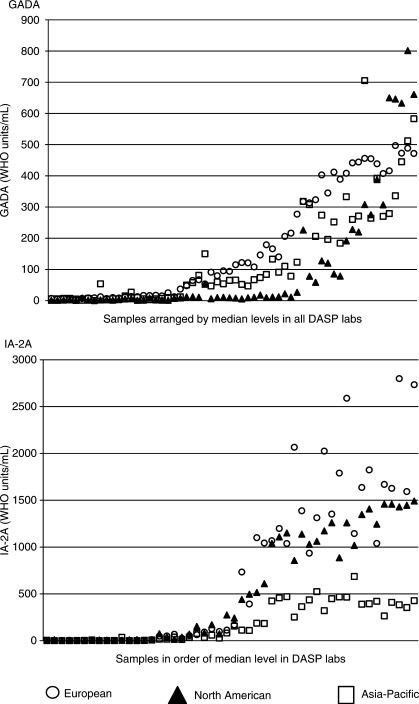
Comparisons of (a) GADA (upper panel) and (b) IA-2A (lower panel) results for cases reported by the three T1DGC autoantibody laboratories in the DASP 2005 workshop. Samples are ordered according to the median antibody level reported by all laboratories participating in DASP 2005.

Antibody levels correlated among laboratories (*p* < 0.0001 for all comparisons). Among the cases, the correlation coefficients (τ) for GADA antibodies were 0.727 (Asia-Pacific vs. North American), 0.744 (European vs. North American), and 0.821 (European vs. Asia-Pacific). For IA-2A antibodies, the correlation coefficients were 0.693 (Asia-Pacific vs. North American), 0.763 (European vs. North American), and 0.687 (European vs. Asia-Pacific). However, as shown in Figure 4(a), the 2005 DASP evaluation demonstrated systematic differences in GADA among the laboratories with lowest GADA antibody levels reported by the North American laboratory (*p* < 0.0001). In contrast, Figure 4(b) shows that the Asia-Pacific laboratory reported the lowest IA-2A levels (*p* = 0.009).

## Discussion

To accomplish a genetics study with a very large number of participants across continents using three different laboratories, the T1DGC set quality control standards for the autoantibody assays to bring the results into the best possible concordance. As part of that process, the T1DGC Study Group reviewed the contemporary protocols and requested efforts to produce similar results for units of islet autoantibody level, requiring the laboratories to examine and contrast their procedures. This communication has summarized the successes and the challenges of the first efforts to achieve those goals.

An initial change was in the use of the IA-2A clone that was considered to be likely to be an important factor in the differences among laboratories. An essential part of this process was active participation of the staff in the laboratories in an iterative exercise in reviewing and harmonizing procedures to achieve much greater concordance among the laboratories.

Ideally, assays are accurate, highly precise, achieve high specificity and sensitivity, and use values that can be reported in standard units that are identical among laboratories and correlate throughout the range of values found in samples. Achieving all of these goals is daunting for any assay, and particularly for islet autoantibody assays in which one is measuring a mixture of different molecules of differing affinity and capacity for islet cell components. Probably no two serum samples (even from the same individual) have identical characteristics and, furthermore, no ‘gold standard’ is available. In this study, we evaluated intra-assay precision, using masked sera, with a split duplicate program and compared quantitative autoantibody units across three laboratories using two different methods for calculating autoantibody levels. With more than 530 masked duplicate samples (sent directly from the clinics, mixed with other samples) assayed concurrently for both GADA and IA-2A, the intra-assay percentage concordance of original positive/negative calls in the three laboratories were 97% and more than 99%, respectively, with excellent correlations overall: *R* = 0.96 for GADA and *R* = 0.99 for IA-2A. Inter-assay variation was not initially assessed, but the later exercise demonstrated good inter-assay reproducibility for these challenging assays over more than 1 year. Accuracy was also not directly assessed given the lack of an independent ‘gold standard method’ to determine actual concentrations of autoantibodies in the sera.

We compared two methods of calculating antibody levels above the highest standard: (1) using an index related to the highest standard and (2) deriving units by extrapolation of the standard curve. Our analysis showed that the mean difference between the pairs was less using the index, indicating that indexes are more reliable for comparing values above the range of the standard curves, at least with these assays.

Within the structure of the DASP proficiency evaluations [6], in which 150 masked samples (50 new onset diabetics mixed with 100 healthy controls) have been tested by all laboratories, we were able to examine differences in assignment of positive/negative status and in quantification of antibody levels using laboratory-defined cutoffs. We found high levels of concordance in positive/negative calls among laboratories, with differences generally occurring only in samples with antibody levels around the threshold. Over the four DASP workshops, each of the laboratories called as many as 7% of controls positive with either the GADA or IA-2A assays at least once, while other laboratories with similar sensitivity reported those samples as negative (Figure 3). Maintenance of specificity along with sensitivity of the assay is obviously crucial as efforts are undertaken to harmonize assays.

A common standard serum sample with defined WHO units/mL (the WHO reference reagent for GADA and IA-2A, 97/550 [11]) was circulated in the DASP workshops and used to calibrate local standards, permitting levels of these autoantibodies in four sets of 150 masked serum samples to be compared among the three T1DGC laboratories. The rankings of samples according to antibody levels were similar among the T1DGC laboratories, but there were systematic differences in the reported antibody levels for both GADA and IA-2A (Figure 4(a) and (b)). Thus, in DASP 2005, using the median results of all participating laboratories as the method of comparison, the North American laboratory reported lower GADA levels in cases than the other two laboratories, suggesting a need to adjust the North American values to align results with those of the other laboratories. Similarly, the lower IA-2A levels reported by the Asia-Pacific laboratory would need adjustment to yield results similar to those reported by the European and North American laboratories. The reasons for the differences have not been fully elucidated, but the laboratories have plans to review and carefully change reagents and protocols within the assays to harmonize the results. Although not proven, the most likely explanations for these differences lie in the different sera used to produce calibrators utilized in each laboratory and in differences in the protocols/materials used (Table 1). In particular, for the GADA assay, the North American laboratory used ^3^H-labeled GADA, while both Asia-Pacific and European laboratories used ^35^S-labeled GADA. For IA-2A, all laboratories used ^35^S-methionine labeling.

There are several caveats for the current study. In particular, we had frequent evaluations of intra-assay variation but only a single inter-assay assessment of technical error. The lack of a common set of standards among the laboratories to minimize long-term drift was a significant impediment to demonstrating long-term consistency among the laboratories. Thus, there is a strong need for a true ‘gold standard’ for all human polyclonal autoantibody assays. Each laboratory had its own program to assess long-term drift, but there were no common T1DGC quality assurance sera to allow this to be externally evaluated.

Leadership in clinical trials and other research studies needs to seek ways to improve the performance of the laboratories producing results. Optimally, a single laboratory performing all analyses should yield consistent results among all samples (assuming the performance of the laboratory remained consistent over time) and could obviate the need to complete the complex comparisons among the laboratories presented in this article. Because the size and complexity of the T1DGC led to the use of separate laboratories on three continents in order to complete the work in a timely manner, we first elected to characterize results as positive/negative, capitalizing on the success of DASP over several years. The T1DGC then initiated quality control measures to ensure robust laboratory performance in testing the consortium samples, using masked split duplicates for 5% of samples collected by clinic sites. Intra-laboratory assay variation was consistently measured, and performance was excellent and sustained. Thus, the quality control procedures have been validated within the limits of the assay. Even with the different procedures used by the laboratories, each laboratory performed adequately.

The opportunity to compare results among the laboratories strengthened efforts to improve the characteristics of the assays by the thorough and critical comparison of results. However, the desirable outcome of reporting identical, quantitative results has been approached, but not achieved in T1DGC. Nevertheless, lessons from the T1DGC emphasize that interactive collegiality and a willingness among the laboratories to cooperate permits maximal harmonization within the limitations of each assay. In our case, the laboratories interacted in a continuous, constructive manner to improve the performances of the assays in each laboratory. Finally, the T1DGC leadership and its sponsors continue to recognize the large amount of effort needed to direct these challenging assays toward much more uniform results among the laboratories.
